# Biogenic mixing induced by intermediate Reynolds number swimming in stratified fluids

**DOI:** 10.1038/srep17448

**Published:** 2015-12-02

**Authors:** Shiyan Wang, Arezoo M. Ardekani

**Affiliations:** 1 University of Notre Dame, Aerospace and Mechanical Engineering, Notre Dame, IN 46556, USA; 2 Purdue University, School of Mechanical Engineering, West Lafayette, IN 47907, USA

## Abstract

We study fully resolved motion of interacting swimmers in density stratified fluids using an archetypal swimming model called “squirmer”. The intermediate Reynolds number regime is particularly important, because the vast majority of organisms in the aphotic ocean (i.e. regions that are 200 m beneath the sea surface) are small (*mm*-*cm*) and their motion is governed by the balance of inertial and viscous forces. Our study shows that the mixing efficiency and the diapycnal eddy diffusivity, a measure of vertical mass flux, within a suspension of squirmers increases with Reynolds number. The mixing efficiency is in the range of *O*(0.0001–0.04) when the swimming Reynolds number is in the range of *O*(0.1–100). The values of diapycnal eddy diffusivity and Cox number are two orders of magnitude larger for vertically swimming cells compared to horizontally swimming cells. For a suspension of squirmers in a decaying isotropic turbulence, we find that the diapycnal eddy diffusivity enhances due to the strong viscous dissipation generated by squirmers as well as the interaction of squirmers with the background turbulence.

Heated and important discussions are rooted in identifying the sources required to sustain ocean mixing. The mechanical power needed to sustain ocean mixing is estimated, using the Levitus climatology, to be more than 2 trillion watts^1^. Mixing across stratification requires a source of kinetic energy^2^. Even though winds and tides are found to be two major mechanical sources for providing the kinetic energy required for ocean mixing, researchers believe that other important sources remain undiscovered^3^.

Mixing can be characterized by the upwelling of water column^1^


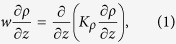


where *w* is the vertical convective velocity along the *z*-direction, *ρ* is the density of the fluid, and *K*_*ρ*_ is the diapycnal eddy diffusivity of density which is a measure of the vertical mass flux. Direct estimates of vertical flux at ocean thermoclines are about one order of magnitude smaller than the bulk’s average value^4^. It suggests that there exist undersampled ‘hot spots’ in the ocean interior at which mixing is much stronger than the values obtained from measurements of vertical flux.

In the ocean interior, the mixing is supplied by small scale mixing events. To date, internal wave breaking generated through winds and abysmal tidal flows are considered to be the major postulated contributors to the interior ocean mixing^2^, however, the direct measurements of mixing in the mid ocean are unreliable^4^. There exists enormous biomass in the ocean interior, which could contribute to mixing. Particularly, a large population of zooplankton and pelagic organisms lives in the mesopelagic region (200–1000 *m* in depth)^5^. Intensive biological activities such as diel vertical migration occur in the mid ocean. The energy imported by biomass is immediately available in the mid ocean while winds and tidal flows import the energy at sea surfaces or topologically complex boundaries. A majority of this energy is dissipated as heat while propagating in the ocean^3^,^5^. Therefore, it is important to examine whether the mixing induced by swimming organisms is important in the ocean interior.

The idea of biogenic mixing was first examined by Munk^6^. The estimated diffusivity using the mixing length argument appeared to be negligible at a large length scale (~300 *m*). However, direct measurements of mixing in the local ‘hot spots’ showed that biogenic mixing could be important. Schooling behavior of marine organisms in an aggregate can produce strong energy dissipation. Huntely and Zhou^7^ first calculated the kinetic energy produced by marine organisms and found the energy dissipation to be on the order of 10^−5^ *W* ⋅ *kg*^−1^, which is 3 to 4 orders of magnitude larger than the average background turbulence in the ocean. Kunze and coworkers measured turbulent energy production in a dense population of euphausiids during the dusk at Sannich Inlet, British Columbia and found it to be on the order of 10^−5^ *W* ⋅ *kg*^−1^^8^. On the other hand, Visser^9^ demonstrated low mixing efficiency in these circumstances because the length scale at which marine organisms import energy is much smaller than the Ozmidov buoyancy length scale in the ocean (~3–10 *m*); he argued that the turbulent kinetic energy of small organisms is mainly dissipated to heat prior to contributing to mixing.

Katija and Dabiri^10^,^11^ suggested mixing via induced drift volume as an alternative mechanism to biogenic mixing: the fluid convects with the migrating body and the mixing will be determined by the drift volume. However, the problem remains unresolved since the drifted volume can re-stratify in marine environments and may not induce mixing. Biogenic mixing cannot be addressed without performing the detailed analysis of swimming organisms in stratified fluids, as it has been done in the recent debate. The present paper provides a fully resolved analysis of the flow generated in a suspension of swimmers interacting in a stratified fluid and quantifies the induced mixing.

Biogenic mixing in a stratified fluid is insignificant when relevant swimming Reynolds number is below unity. Wagner *et al*.^12^ estimated the mixing efficiency for swimming microorganisms using the solution of point force singularities in stratified fluids given by Ardekani and Stocker^13^ and found the biogenic mixing to be minute when the inertial effects are neglected. Kunze^14^ estimated the diapycnal eddy diffusivity *K*_*ρ*_ through scaling analysis and found it to be negligible for *Re* = *Ua*/*ν* < 1, where *U* is the characteristic swimming velocity of the organism, *a* is the body size of the organism and *ν* is the kinematic viscosity of the fluid. In the aphotic ocean (i.e. regions that are 200 m beneath the sea surface), zooplankton are the most abundant organisms leading to vertical fluid transport^6^,^15^, and their body size ranges from millimeters to centimeters. The corresponding swimming Reynolds number is in the range of *Re* ~ *O*(1–100). Recent experimental studies have shown that swimming in the inertial regime generates enhanced dissipation rate whose spatial scale exceeds organisms’ body size in the absence^15^,^16^ and presence of the density stratification^17^. Therefore, it is important to examine the hydrodynamic interaction of swimming organisms in this inertial regime and to quantify the induced mixing. In this manuscript, we study fully resolved motion of interacting swimmers in density stratified fluids using an archetypal swimming model called “squirmer”. Further, we consider a suspension of squirmers in a decaying isotropic turbulence, resembling the scenario where marine organisms hydrodynamically interact in the presence of the background turbulence.

## Numerical Framework

We performed a fully-resolved three dimensional direct numerical simulation of a suspension of squirmers. Although the squirmer model was originally proposed to study low Reynolds number swimming^18^,^19^, it has been recently extended to the inertial regime^20^,^21^,^22^. It effectively models the flow generated by a coordinated beating of cilia on the surface of organisms, such as ctenophora, living worldwide in marine environments^23^. Their sizes range from millimeters to centimeters, and their Reynolds number is on the order of *O*(10^2^). For a spherical squirmer, an axisymmetric tangential surface velocity is given as^18^


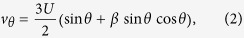


where *θ* is the polar angle measured from the swimming direction, *β* scales with *aω*/*U*, *ω* is the vorticity generated by the squirmer, *U* and *a* are the characteristic swimming velocity and the radius of the squirmer, respectively. For example, *aω*/*U* for the copepod nauplii (*Temora longicornis*) is about two in a swimming mode (*ω* ~ 70 *s*^−1^, *a* ~ 300 *μm*, *U* ~ 10^−2^ *m* ⋅ *s*^−1^)^24^. *β* is positive (negative) for the organisms generating trust in front of (behind) their body called puller (pusher). The puller brings fluid from front and back and expels it from the side, while pusher does the opposite. The flow generated by a squirmer in a stratified fluid strongly affects the density field and consequently, the squirmer’s motion^25^. Here, we investigate the mixing induced by a suspension of squirmers in stratified fluids in the absence and presence of the background turbulence. We should note, however, that the squirmer is only a reduced-order model for the locomotion of organisms. This reduced-order squirmer model has been broadly employed to examine various aspects of swimming at low Reynolds number, such as hydrodynamic interaction of two organisms^26^, swimming near a wall^19^, suspension dynamics^27^, and optimal feeding^28^.

The governing equations for an incompressible viscous fluid under Boussinesq approximation are given as


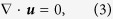



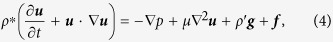



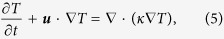


where ***u*** = (*u*, *v*, *w*) is the flow velocity, 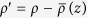 is the density perturbation from the linear background density profile 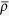, *p* is the disturbance pressure, *μ* is the dynamic viscosity of the fluid, 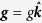 is the gravitational acceleration, 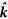 is the unit vector along the direction of gravity, *T* is the temperature, and *κ* is the thermal diffusivity. The body force ***f*** in Equation (4) accounts for the hydrodynamic effect imposed by freely swimming squirmers^29^. The density *ρ* and ρ^*^ can be written as *ρ* = *ρ*_*f*_ + (*ρ*_*p*_ − *ρ*_0_)Φ and ρ^*^ = *ρ*_0_ + (*ρ*_*p*_ − *ρ*_0_)Φ, respectively, where *ρ*_*f*_ is the fluid density, *ρ*_0_ is the volume-averaged density of the fluid, and *ρ*_*p*_ is the squirmer density. The phase indicator parameter Φ is unity inside the squirmer and zero elsewhere in the computational domain. Equations (3, 4, 5) are derived from the conservation of mass, momentum, and energy for an incompressible fluid, respectively. The density variation across thermocline occurs due to the vertical variation in temperature, 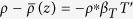, where *β*_*T*_ is the thermal expansion coefficient, 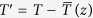 is the temperature perturbation and 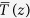 is the linear background temperature. The diffusivity coefficients are assumed to be uniform and the same for the squirmer and the background fluid^30^. In order to obtain a steady-state condition, we set 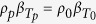, where 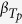 and 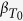 correspond to the thermal expansion coefficient of the squirmer and background fluid, respectively. Equation (5) can be rewritten in terms of temperature perturbation *T*′ as


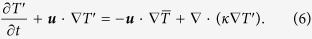


The density of the background fluid linearly changes with temperature, which is an appropriate assumption for centimeter size organisms. The magnitude of the stratification can be characterized by the Brunt-Väisälä frequency, 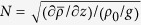. Considering both mild and strong density gradients in the ocean, the value of *N* is around 10^−4^ − 0.3 *s*^−1^^31^,^32^. The dynamics of swimming in a stratified fluid can be characterized by three independent dimensionless parameters. The buoyancy effects can be characterized by Froude number *Fr* = *U*/(*Na*). Reynolds number *Re* = *Ua*/*ν* characterizes the ratio of inertial to viscous forces. The Prandtl number *Pr* = *ν*/*κ* measures the ratio of the momentum diffusivity *ν* to the thermal diffusivity *κ*, and its value is about 7 for temperature stratified fluids. Equations (3), (4), and (6) are solved numerically, the details of which are given in the ‘Methods’ section. Unless otherwise stated, squirmers are force-free, torque-free and neutrally buoyant (*ρ*_*p*_ = *ρ*_0_) and they are initially oriented along the gravitational direction. The simulations are continued till the entire system reaches quasi-steady state when both kinetic energy and temperature perturbation satisfy^12^


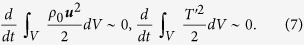


## Results

We performed simulations for a swarm of pushers and pullers in the range of Reynolds number between 0.1 and 100 in a linearly stratified fluid in the absence and presence of background turbulence. The biogenic mixing process is quantified by calculating the mixing properties, such as mixing efficiency, diapycnal eddy diffusivity, and Cox number.

Huntley and Zhou^7^ have provided the empirical relationship for the packing density of the organisms in the ocean. The volume fraction for escaping mode *ϕ*_*e*_ and cruising mode *ϕ*_*c*_ can be estimated as


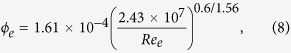



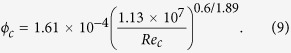


The estimated volume fraction of organisms swimming at Re = 10 is *ϕ*_*e*_ = 4.6% and *ϕ*_*c*_ = 1.34%. The volume fraction of marine organisms at an intermediate Reynolds number rarely exceeds 4%^7^. In this study, unless otherwise stated, we consider a volume fraction of 4% to obtain an upper bound of the biogenic mixing. Hydrodynamic interactions at this volume fraction, corresponding to a semi-dilute regime (<10%), are mainly pairwise^27^. Consequently, small number of squirmers can accurately capture the physics of the problem.

### Mixing efficiency

**Figure 1:**
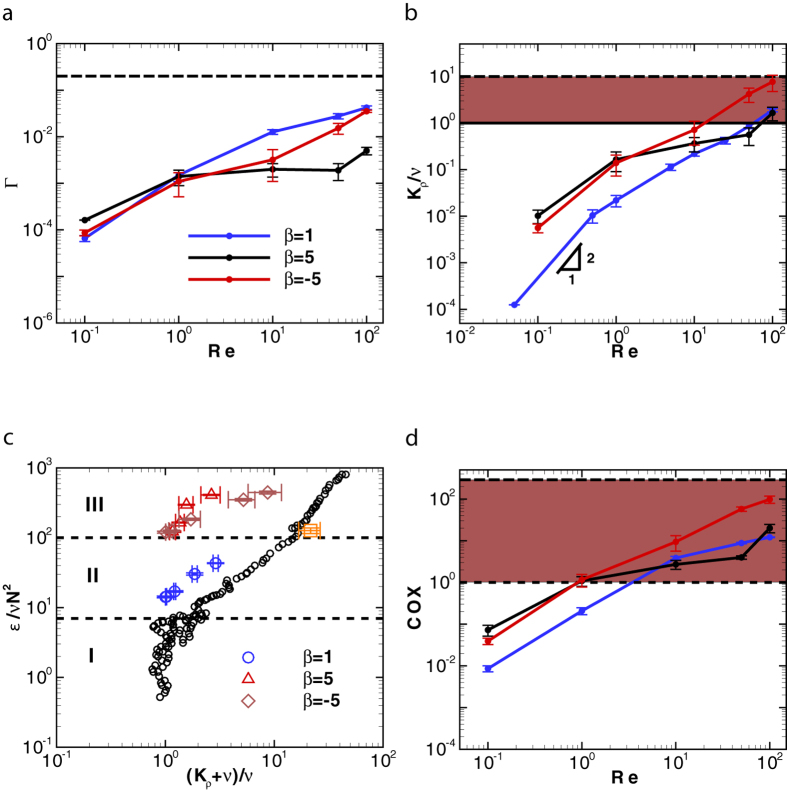
The effect of inertia on the biogenic mixing is quantified. (**a**) Mixing efficiency generated by a swarm of squirmers is plotted for different Reynolds numbers; the horizontal dashed line represents the mixing efficiency for a typical turbulent mixing, Γ = 0.17. (**b**) the normalized diapycnal eddy diffusivity is plotted for different Reynolds numbers; the horizontal solid line represents the value of kinematic viscosity (momentum diffusivity); the horizontal dashed line represents the diapycnal eddy diffusivity caused by the internal wave breaking in the ocean^14^. (**c**) The normalized kinetic dissipation between the shear-driven turbulence^34^ (black circles) and mixing generated by squirmers are compared. The orange square corresponds to our calculation of turbulent mixing. (**d**) The Cox number is plotted for different Reynolds numbers; the dotted dashed and long dashed lines correspond to the measured minimum and maximum values of Cox number at North Pacific thermocline^36^, respectively. The data points are time-averaged values after the system has reached quasi-steady state; the error bars represent the temporal standard deviation of the time-averaged quantity. The volume fraction is *ϕ* = 4%, and *Fr* = 5.29.

For a swarm of squirmers in a linearly stratified fluid, the kinetic energy equation, in a quasi-steady state, is written as


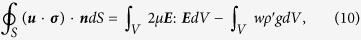


where *S* represents the squirmers’ surfaces, *n* is the unit vector normal to the surface *S*, *V* is the entire fluid domain, and ***E*** is the strain rate tensor. The term on the left hand side of equation (10) is the total energy input generated by squirmers. The first and second terms on the right hand side of equation (10) represent the viscous dissipation and the rate of creation of gravitational potential energy in the entire fluid domain, respectively. The energy input is generated due to the fluid stress ***σ*** at the surface of the squirmers. Consequently, the disturbance induced by the freely moving squirmers is the main source of mechanical energy input to the surrounding fluid. On the other hand, turbulent mixing imports mechanical energy through the turbulent production term. For example, in a shear turbulence, the turbulent production term is 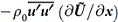, where 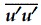 is the turbulent Reynolds stress tensor and ∂*Ũ*/∂x is the mean shear of the turbulence. The efficiency of turbulent mixing, also called the flux Richardson number, has been defined as the ratio of the rate of removal of energy by the buoyancy forces to the total turbulent energy production^33^,^34^. The total energy input balances the viscous dissipation and the buoyancy flux of the fluid. The efficiency Γ for a biogenic mixing process is defined as


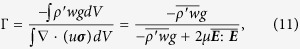


where overbar denotes the averaged quantities over the entire fluid domain. Wagner *et al*. utilized equation (11) to estimate the mixing efficiency induced by a single micron-size microorganism which is on the order of 10^−8^ in an ocean environment^12^. The analytic solution was derived using solution of point force singularities in a stratified fluid^13^ for a visco-diffusive regime, where the inertial effects are neglected. For a suspension of squirmers in a linearly stratified fluid, we find that the mixing efficiency increases with inertia (Fig. 1a). For 10 < *Re* < 100 and |*β*| = 5, the mixing efficiency achieved by pushers is larger than pullers. The largest mixing efficiency produced by squirmers is (0.042 ± 0.0036) for *Re* = 100, *β* = 1 and *Fr* ~ 5.2 which is a strong stratification (deeper estuaries have a typical value of *N* ~ 0.1^32^, leading to a Froude number in the range of 5 < *Fr* < 30). This value is smaller than the mixing efficiency in a typical turbulent mixing event (Γ ~ 0.17)^33^.

### Diapycnal eddy diffusivity

The diapycnal eddy diffusivity of density is defined as^31^,^33^


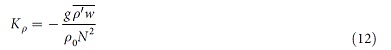


and quantifies the mixing due to vertical transport^34^. Kunze^14^ showed that *K*_*ρ*_ scales as (*K*_*ρ*_/*ν*) ~ *γ*_1_*Re*^2^*ϕ*, where *γ*_1_(<1) is the correlation coefficient and *ϕ* is the volume fraction of the organisms in an aggregate. The eddy diffusivity obtained from the present simulations agrees well with Kunze’s scaling for *Re* < 1 and *β* = 1 (blue line in Fig. 1b). The diapycnal eddy diffusivity for *Re* < 1 and *β* = ±5 (−5 and +5 shown with black and red lines, respectively in Fig. 1b) deviates from Kunze’s formula due to the importance of hydrodynamic interactions between squirmers with large |*β* | generating strong vorticity. Therefore, the scaling analysis cannot capture the biogenic mixing generated by a suspension of squirmers even at low values of Reynolds numbers. The diapycnal eddy diffusivity increases with inertia. For *Re* > 10 (Fig. 1b), pushers generate more effective mixing than pullers and the magnitude of diapycnal eddy diffusivity at *Re* = 50 and *β* = −5 exceeds that of the molecular diffusion. Noss and Lork^35^ experimentally evaluated the vertical mass flux generated by a freely swimming *Daphina* (*Re* ~ 50, *a* ~ 0.5 *mm*, *U* ~ 1 *cm* ⋅ *s*^−1^, *ϕ* ~ 1%) in a density stratified fluid, and showed its value to be about 0.8 × 10^−5^ *m*^2^ ⋅ *s*^−1^ which is consistent with our numerical results for pushers swimming at *Re* = 50. At *Re* = 100 which resembles swimming of centimeter-sized organisms, the mixing is comparable to small scale turbulent mixing caused by internal wave breaking (10^−5^ *m*^2^ ⋅ *s*^−1^), a major contributor to the interior ocean mixing^2^,^14^.

It would be illustrative to compare the values of diapycnal eddy diffusivity due to biogenic mixing to those generated by turbulent events. For shear turbulence in a stratified fluid, three distinctive regions can be identified based on the turbulent activity parameter *ε*/*νN*^2^^34^, the ratio of the destabilizing effect of turbulent stirring to the stabilizing effects originated from the combination of buoyancy and viscosity. Here, *ε* = 2*ν**E*** : ***E***. Strong mixing occurs for large values of *ε*/*νN*^2^. Region I in Fig. 1c corresponds to the decaying turbulence in a stratified fluid for *ε*/*νN*^2^ < 7; region II corresponds to the stationary turbulence which occurs when 7 < *ε*/*νN*^2^ < 100; region III is associated with growing turbulence which occurs for *ε*/*νN*^2^ > 100^34^. Figure 1c shows that the majority of data points representing the biogenic mixing induced by squirmers lie above the scaling curve for the turbulent mixing. The dissipation generated by the squirmers in a stratified fluid corresponds to regions II and III. The mixing generated by the squirmers in local hot spots can generate eddy diffusivities as strong as turbulent mixing, but it occurs at larger values of energy dissipation compared to turbulent mixing.

### Temperature microstructure

To quantify the local temperature microstructure at thermocline, the Cox number^31^,^36^ is used and is defined as


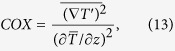


where 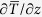 is the mean temperature gradient. The Cox number provides a measure of the variance of the temperature gradient in the fluid. Gregg^36^ has reported the seasonal Cox number to be between 1 and 290 at North Pacific thermocline. For the mixing generated by the squirmers at an intermediate Reynolds number, the temperature microstructure is generated through the combination of the squirmer’s locomotion and the thermal diffusion of the fluid. The Cox number in a swarm of squirmers can be as large as *O*(100) (see Fig. 1d).

### Effect of swimming orientation

Unless otherwise stated, the squirmers are initialized to swim in the vertical direction; however, their swimming orientation evolves over time due to their hydrodynamic interaction, collision as well as the background turbulence because they are modeled as force-free, torque-free swimmers. We have performed an additional simulation where the swimmers are constrained to swim in the vertical direction by applying an external torque. In this case, the mixing efficiency, diapycnal eddy diffusivity, and Cox number enhance for vertically swimming pushers (*β* = −5, *ϕ* = 4%, Re =50, *Fr* = 5.29) by a factor of 1.72, 1.88, and 3.00, respectively, compared to force-free, torque-free swimmers. The mixing efficiency, diapycnal eddy diffusivity, and Cox number enhance by a factor of 7.43, 7.98 and 8.26 for pullers (*β* = 5, *ϕ* = 4%, Re =50, *Fr* = 5.29), respectively.

**Figure 2:**
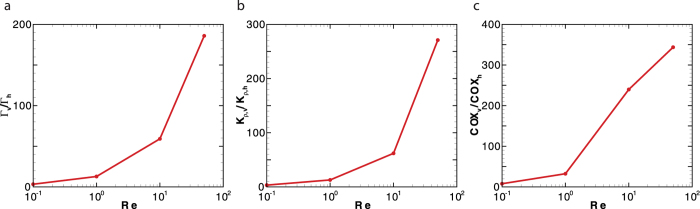
A comparison of mixing induced by vertically moving squirmers in a regular array to horizontally moving squirmers in terms of (a) mixing efficiency, (b) diapycnal eddy diffusivity, and (c) Cox number. The corresponding dimensionless parameters are *Fr* = 5.29, *ϕ* = 4%, and *β* = 1. All the data points represent the ratio of quasi-steady state values for vertically swimming cells to horizontally swimming cells.

The mixing induced by squirmers is sensitive to the initial swimming orientation *α*, measured from the gravitational direction. For a regular array of squirmers, the ratio of mixing efficiency for vertically swimming cells (*α* = 0°) to horizontally swimming cells (*α* = 90°) increases with Reynolds number (see Fig. 2a). We should note that a regular array refers to a single squirmer in a periodic computational domain. Thus, the swimming orientation remains constant except for large enough Reynolds number (*Re* ≥ 10) where inertial effects lead to instability in the swimming orientation of a puller. For *Re* < 1, this ratio is below 10, which is close to the analytical results in a zero-Reynolds-number regime^12^. The distinction between values of both diapycnal eddy diffusivity and Cox number for vertically and horizontally swimming cells significantly increases with Reynolds number (see Fig. 2b,c).

### Effect of system size and volume fraction

**Figure 3:**
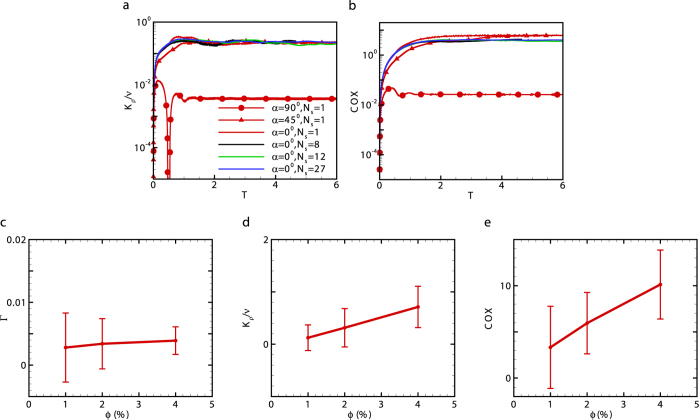
The effect of domain size and initial swimming orientation of squirmers on (a) the diapycnal eddy diffusivity and (b) Cox number is plotted for a swarm of pullers (*β* = 1) with a volume fraction of *ϕ* = 4%. The effects of volume fraction of pushers (*β* = −5) are shown on the (**c**) mixing efficiency, (**d**) eddy diffusivity and (**e**) temperature microstructure. The corresponding dimensionless numbers are *Re* = 10 and *Fr* = 5.29. Data points in Figure (**c**), (**d**), (**e**) represent the time-averaged values after the system has reached quasi-steady state; the error bar represents the temporal standard deviation of the time-averaged quantity.

For a given volume fraction, the effect of the system size has been tested for different number of squirmers, *N*_*s*_ = 1, 8, 12, 27. The temporal evolution of the diapycnal eddy diffusivity *K*_*ρ*_ and Cox number are shown in Fig. 3a,b, where the dimensionless time is calculated as *T* = *tν*/*a*^2^. The results are independent of the system size for *N*_*s*_ ≥ 8^37^. Unless otherwise stated, we use *ϕ* = 4% and *N*_*s*_ = 8.

For a given system size, the effect of volume fraction has been evaluated by changing the number of squirmers in the computational domain. The mixing efficiency is almost independent of the volume fraction (Fig. 3c). Both the diapycnal eddy diffusivity and Cox number increase with the volume fraction (Fig. 3d,e).

### Effect of density stratification

**Figure 4:**
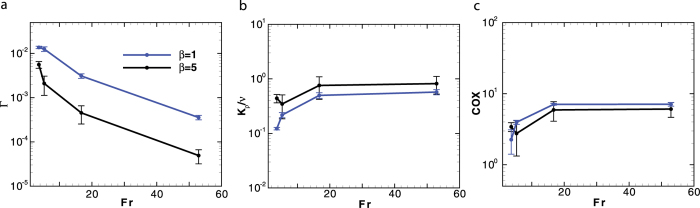
The effect of fluid stratification on the biogenic mixing is quantified. (**a**) The mixing efficiency decreases with the Froude number; (**b**) The normalized diapycnal eddy diffusivity and (**c**) the Cox number are independent of the Froude number for *Fr* > 20. The corresponding dimensionless parameters are *Re* = 10 and *ϕ* = 4%. All data points represent the time-averaged values after the system has reached quasi-steady state; the error bar represents the temporal standard deviation of the time-averaged quantity.

Density stratification strongly affects the mixing efficiency generated by a suspension of squirmers in a stratified fluid. The mixing efficiency decreases with the Froude number (see Fig. 4a). For *Re* = 10, the change of the mixing efficiency is about two orders of magnitude from *Fr* = 3.6 to 52.9. On the other hand, the overall vertical mass flux and temperature microstructures are nearly independent of the density stratification for *Fr* > 20. The values of diapycnal eddy diffusivity (Fig. 4b) and Cox number (Fig. 4c) slightly increase at small *Fr* (*Fr* < 20). It has been noted by Ardekani and Stocker^13^ that the fundamental length scale of the stratification in aquatic environments is 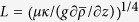. When stratification length scale is larger than the swimmer size, i.e., *L*/*a* ~ 1, the behavior is the same as the homogeneous-density fluid and is independent of stratification (*L*/*a* ~ 1 corresponds to *Fr* ~ 30). The local shear generated by swimming ctenophores is about *U*/*a* ~ 0.5*s*^−1^^23^, which corresponds to *Fr* ~ 50 (*N* ~ 0.01). Therefore, the vertical mass flux induced by organisms swimming across weak stratification is similar to the one in a homogenous fluid. On the contrary, the fluid stratification strongly reduces the turbulent mixing. The diapycnal eddy diffusivity of the turbulent mixing scales as *ε*/(*νN*^2^) (see black circles in Fig. 1c). The dissipation of kinetic energy in a turbulent flow scales as *ε* ~ *u_t_*^3^/*l* where *u*_*t*_ and *l* are the characteristic velocity and the integral length scale of the turbulence, respectively. Thus, the turbulent activity parameter can be written as 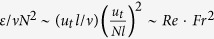. Consequently, turbulent mixing is suppressed in the region of strong vertical density gradient (e.g. pycnoclines), unlike mixing induced by swimming organisms.

### Effect of buoyancy

**Figure 5:**
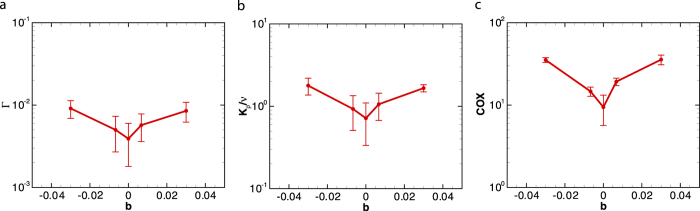
The effect of buoyancy on the (a) mixing efficiency, (b) diapycnal eddy diffusivity, and (c) Cox number generated by a swarm of pushers (*β* = −5). The corresponding dimensionless numbers are *Re* = 10 and *Fr* = 5.29. All Data points represent the time-averaged values after the system has reached quasi-steady state; the error bar represents the temporal standard deviation of the time-averaged quantity.

Here, we consider the motion of negatively (*ρ*_*p*_ > *ρ*_0_) and positively (*ρ*_*p*_ < *ρ*_0_) buoyant swimming organisms. Figure 5 shows the role of buoyancy on the mixing efficiency, diapycnal eddy diffusivity and Cox number for *Re* = 10, *Fr* = 5.29, *ϕ* = 4%, *β* = −5. The squirmer’s buoyancy is characterized by *b* = (*ρ*_*p*_ − *ρ*_0_)/*ρ*_0_. The values of *b* = 0.0067 and *b* = 0.03 are selected based on the reported excess density for copepod relative to the ambient fluid which is in the range of 6.7 kg ⋅ m^−3^ to 30 kg ⋅ m^−3^^38^,^39^. Mixing parameters are enhanced for both positively and negatively buoyant swimmers compared to a neutrally buoyant case (*b* = 0).

### Effect of marine turbulence

Turbulent flows are prevalent in marine environments. Therefore, it is necessary to investigate how the organisms interact with the background turbulence^40^. We will show that the biogenic contribution to the total mixing is determined by the magnitude of dissipation of kinetic energy introduced by the organisms. Even though Gregg and Horne’s^41^ studies were designed to answer this question, isolation of the effects of the organisms’ swimming from turbulence is difficult, if not impossible, in a natural environment. Direct numerical simulations performed here allow us to separately consider both effects of turbulence and flow disturbances generated by squirmers. We investigate the hydrodynamic interaction of a suspension of swimmers in a decaying stratified turbulence. The initial stratified turbulent flow is generated using direct numerical simulations with a direct forcing approach^42^,^43^, the details of which have been elaborated in the ‘Methods’ section. The Taylor Reynolds number for the turbulence is *Re*_*λ*_ = *u*_*rms*_*λ*/*ν* ~ 49 and *ε*/(*νN*^2^) ~ 126, where *u*_*rms*_ is the root-mean-square of the turbulent velocity and *λ* is the Taylor length scale. The corresponding mixing parameters are calculated as *K*_*ρ*_/*ν* = 20.56 ± 4.82, *COX* = 149.28 ± 29.87, and Γ = 0.139 ± 0.027. The value of diapycnal eddy diffusivity agrees well with the curve suggested by Shih *et al*.^34^ (see hollow orange square in Fig. 1c). Gregg and Horne took modular microstructure profilers (MMPs) for measuring temperature and velocity microstructure within and outside aggregates of organisms^41^, and relevant mixing properties of our turbulence simulation are close to one of their MMPs (MMP15041) outside the aggregate (*K*_*ρ*_ = 1.71 × 10^−5^ *m*^2^/*s*, *COX* = 120, Γ = 0.101). The squirmers’ size is about *a* ~ 1.07*λ* and *a* ~ 14.69*η*, where *η* is the kolmogorov length scale. The squirmer Reynolds number is *Re* = *Ua*/*ν* = 100, representing swimming of marine organisms of centimeter size and |*β*| = 3.

To quantify the relative extent of the biogenic mixing (in the absence of background flow) with regards to the turbulent mixing, a “biomixing active parameter” is defined as


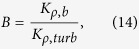


where subscripts *b* and *turb* refer to biogenic mixing and turbulent mixing, respectively. The diapycnal eddy diffusivity can be expressed in terms of mixing efficiency Γ, and equation (12) simplifies as


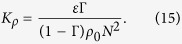


For biomixing, Γ_*b*_ ≪ 1^9^,^41^ and equation (15) is simplified as *K*_*ρ*_ = Γ_*b*_*ε*_*b*_/(*ρ*_0_*N*^2^). For general turbulent mixing in marine environments, Γ_*turb*_ ~ 0.17 and *K*_*ρ*_ ~ 0.2*ε*_*turb*_/(*ρ*_0_*N*^2^)^33^. Therefore, biomixing active parameter can be expressed as


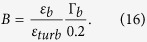


**Figure 6:**
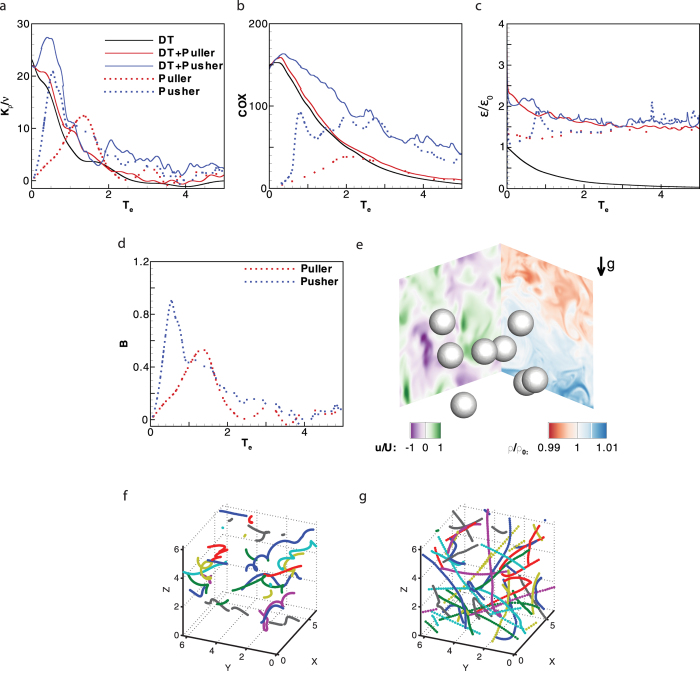
A decaying stratified turbulence is modulated by squirmers of Taylor length-scale size. The transient behavior of the overall mixing is described by the temporal evolution of (**a**) the normalized diapycnal eddy diffusivity, (**b**) Cox number and (**c**) normalized dissipation of kinetic energy. (**d**) A dynamic evolution of *B* is shown for both pullers and pushers. (**e**) A snapshot of a suspension of 8 pushers is shown at *T*_*e*_ = 0.2 (supplementary movie 1 shows swimming dynamics of pushers). (**f**,**g**) correspond to trajectories of pullers and pushers, respectively, recorded over 4*T*_*e*_ where different colors distinguish individual squirmers (supplementary movie 2 and 3 show swimming trajectory of pushers and pullers, respectively). The corresponding dimensionless numbers are |*β*| = 3 and *ϕ* = 2%. ‘DT’ corresponds to flow with decaying turbulence; ‘DT + Puller’ and ‘DT + Pusher’ correspond to a suspension of pullers and pushers swimming in a decaying turbulence, respectively; ‘Puller’ and ‘Pusher’ correspond to a suspension of pullers and pushers in the absence of turbulence.

In our simulations, the temporal evolution of biomixing active parameter *B* is calculated for a suspension of squirmers, and its maximum value is about 0.9 (see Fig. 6d).

The results reveal that pushers more strongly affect the overall mixing compared to pullers. For a decaying stratified turbulence without squirmers (black solid line in Fig. 6a), normalized *K*_*ρ*_/*ν* is suppressed within two eddy turnover times, where *T*_*e*_ = *t*/*τ*_0_ is the normalized time scale and *τ*_0_ is the initial eddy turnover time of the turbulence. With the entrainment of the squirmers of Taylor length-scale size, the dynamics of mixing can be characterized by two distinct stages. In stage I, the value of *B* for both pushers and pullers is above 0.2 (see Fig. 6d), due to the hydrodynamic interactions between the turbulence and the squirmers’ disturbances. In stage II, the turbulent structure decays away and the flow that is induced by the squirmers is strong. During stage I when *T*_*e*_ < 2, pushers (blue solid line in Fig. 6a) significantly affect the mixing, while the pullers (red solid line in Fig. 6a) do not affect the mixing generated by the decaying turbulence. This is consistent with the results of squirmers in a quiescent flow where pushers (red dotted line in Fig. 6a) are able to generate larger mixing in a stratified fluid than pullers (blue dotted line in Fig. 6a). The dissipation of kinetic energy is also larger for pushers compared to pullers, where *ε*/*ε*_0_ in Fig. 6c is the normalized dissipation of kinetic energy and *ε*_0_ is the initial dissipation of the decaying turbulence. Similarly, pushers (blue solid line in Fig. 6b) strengthen temperature microstructures in a stratified decaying turbulence, as shown by the dramatic increase of the Cox number compared to the case corresponding to the decaying turbulence in the absence of swimmers (black solid line in Fig. 6b). During stage II, the fine structure of the turbulence decays away. Both the eddy diffusivity and Cox number decrease to their values for a suspension of squirmers in a quiescent flow.

The strong mixing of fluids generated by pushers compared to pullers can be explained by their swimming trajectories. Pushers (Fig. 6g) rectilinearly swim with infrequent changes in their swimming direction due to the squirmer-squirmer interactions, while pullers (Fig. 6f) swim in helical pathes. Even though the slip velocity is axisymmetric for both pushers and pullers, pullers swim in helical trajectories due to their hydrodynamic interactions as well as inertial effects. Our previous results^44^ show that even a single puller at large enough Reynolds number becomes unstable and will not swim on a straight line due to inertial effects. This is not the case for a pusher in the range of Reynolds number investigated in this work. Our previous results for two squirmers moving toward each other in a relatively large computational domain^44^ shows that pullers move on circular trajectories after the collision, whereas swimming direction of a pusher does not change. Over a duration of 4*T*_*e*_, pushers are much more dispersed compared to pullers (see Fig. 6f and 6g). Consequently, pushers create larger disturbances in stratified fluids. The extent of biogenic mixing depends on the swimming mode of organisms during their migration.

## Conclusions

We have numerically studied the hydrodynamic interactions of swimmers with each other, background stratification as well as turbulence. For swimming at *Re* < 1, the diapycnal eddy diffusivity is generally below the value of molecular diffusion. Therefore, the biogenic mixing at low inertial regime is negligible. Our results show that the mixing efficiency, diapycnal eddy diffusivity, and temperature microstructure increase with inertia. The mixing efficiency induced by vertical swimming can be two orders of magnitude larger than horizontal swimming. Vigorous kinetic dissipation generated by a suspension of swimmers enhances the mass transport as well as the temperature microstructure in the presence of background turbulence. Pushers induce larger mixing compared to pullers, which is due to their rectilinear swimming behaviour as opposed to helical motion of pullers. The values of diapycnal eddy diffusivity and Cox number at Re~O(100) are on the same order as the ones caused by turbulent mixing.

## Methods

### Modeling squirmers in a quiescent background flow

The simulations are conduced in a fixed frame of reference. Equations (3), (4), and (6) are solved in a cubic box of 2*π* × 2*π* × 2*π*. The boundary conditions for ***u***, *p*, and *T*′ are periodic in all three directions. Convection and diffusion terms in Equation (4) are discretized using QUICK (Quadratic Upstream Interpolation for Convective Kinetics)^45^ and central difference schemes, respectively. Both convection and diffusion terms in equation (6) are solved using central difference schemes to conserve perturbation temperature *T*′^2^ in space. The temporal discretization is performed using the first-order forward Euler method. The projection method is utilized to enforce the continuity condition in equation (3). The resultant Poisson equation for the pressure is solved using the Hypre library^46^. The tangential squirming motion *v*_*θ*_ on the surface of the spherical particle is satisfied by using a distributed Lagrange multiplier (DLM) technique as explained in our previous publication^29^,^47^. The DLM technique has been broadly utilized to study a suspension of inert particles^37^ as well as squirmers^19^,^29^. A short-range repulsive force is used to model squirmer-squirmer collision, the details of which are given in our previous work^29^.

### Modeling background turbulence

Turbulence ubiquitously occurs in marine environments. In order to study the hydrodynamic interactions between the turbulence and the disturbances induced by squirmers, a statistically steady stratified turbulence is numerically generated and treated as the initial background flow. Equations (3), (4), and (6) are solved. Instead of calculating the body force using the DLM technique, however, ***f*** is a direct forcing term which is given as 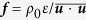. The initial temperature field linearly varies with depth. The initial velocity field is generated using an open source code (NTMIX-3D) provided by Centre de Recherche sur la Combustion Turbulente^48^. The initial velocity profile has the following energy spectrum *E*(***k***) suggested by Passot and Pouquet (1987)^49^,


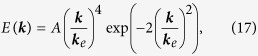


where ***k*** is the wave number, ***k***_*e*_ represents the most energetic wave number and 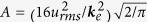. The stratified turbulence reaches a statistically stationary state as both 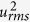 and *ε* reach a quasi-steady value^43^. Once the statistically stationary state is achieved, the direct forcing is no longer required and the stratified turbulence decays. The kolmogorov scale is well resolved in the simulations and *ηk*_*max*_ = 5.23 > 1, where *k*_*max*_ = *πN*_*g*_/*L*, *N*_*g*_ = 256 is the number of grid points, and *L* = 2*π* is the length of the computational domain.

## Additional Information

**How to cite this article**: Wang, S. and Ardekani, A. M. Biogenic mixing induced by intermediate Reynolds number swimming in stratified fluid. *Sci. Rep*. **5**, 17448; doi: 10.1038/srep17448 (2015).

## Supplementary Material

Supplementary Movie 1

Supplementary Movie 2

Supplementary Movie 3
