# Supplementary figures and images for: Biogenic mixing induced by intermediate Reynolds number swimming in stratified fluids
Source: Sci Rep. 2015 Dec 2;5:17448. doi: 10.1038/srep17448 (PMC4667182; doi:10.1038/srep17448)

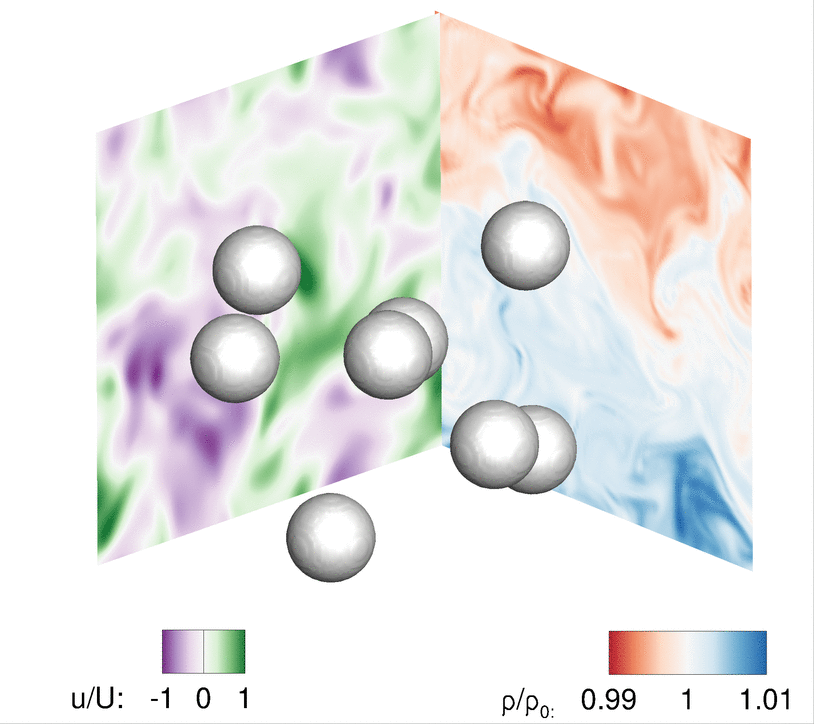

Supplement: Supplementary Movie 1 [file srep17448-s1.gif]

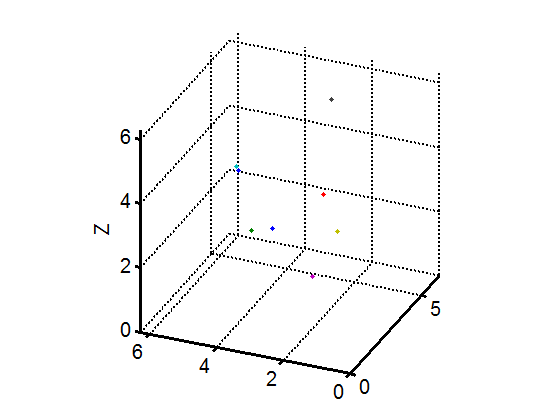

Supplement: Supplementary Movie 2 [file srep17448-s2.gif]

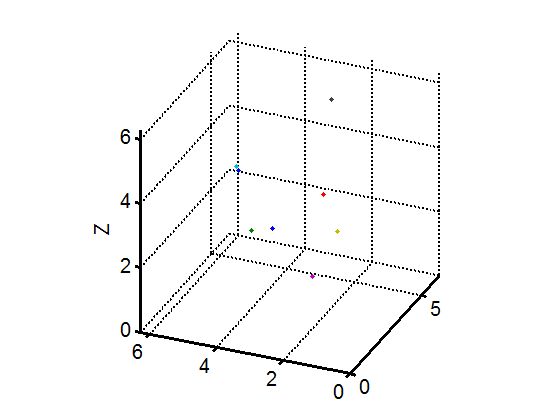

Supplement: Supplementary Movie 3 [file srep17448-s3.gif]
